# Modeling microelectrode biosensors: free-flow calibration can substantially underestimate tissue concentrations

**DOI:** 10.1152/jn.00788.2016

**Published:** 2016-12-02

**Authors:** Adam J. H. Newton, Mark J. Wall, Magnus J. E. Richardson

**Affiliations:** ^1^Warwick Mathematics Institute, University of Warwick, Coventry, United Kingdom; and; ^2^School of Life Sciences, University of Warwick, Coventry, United Kingdom

**Keywords:** biosensors, calibration, modeling

## Abstract

Microelectrode amperometric biosensors are widely used to measure concentrations of analytes in solution and tissue including acetylcholine, adenosine, glucose, and glutamate. A great deal of experimental and modeling effort has been directed at quantifying the response of the biosensors themselves; however, the influence that the macroscopic tissue environment has on biosensor response has not been subjected to the same level of scrutiny. Here we identify an important issue in the way microelectrode biosensors are calibrated that is likely to have led to underestimations of analyte tissue concentrations. Concentration in tissue is typically determined by comparing the biosensor signal to that measured in free-flow calibration conditions. In a free-flow environment the concentration of the analyte at the outer surface of the biosensor can be considered constant. However, in tissue the analyte reaches the biosensor surface by diffusion through the extracellular space. Because the enzymes in the biosensor break down the analyte, a density gradient is set up resulting in a significantly lower concentration of analyte near the biosensor surface. This effect is compounded by the diminished volume fraction (porosity) and reduction in the diffusion coefficient due to obstructions (tortuosity) in tissue. We demonstrate this effect through modeling and experimentally verify our predictions in diffusive environments.

**NEW & NOTEWORTHY** Microelectrode biosensors are typically calibrated in a free-flow environment where the concentrations at the biosensor surface are constant. However, when in tissue, the analyte reaches the biosensor via diffusion and so analyte breakdown by the biosensor results in a concentration gradient and consequently a lower concentration around the biosensor. This effect means that naive free-flow calibration will underestimate tissue concentration. We develop mathematical models to better quantify the discrepancy between the calibration and tissue environment and experimentally verify our key predictions.

## Abstract (precis)

Microelectrode biosensors are typically calibrated in a free-flow environment where the concentrations at the biosensor surface are constant. However, when in tissue, the analyte reaches the biosensor via diffusion and so analyte breakdown by the biosensor results in a concentration gradient and consequently a lower concentration around the biosensor. This effect means that naive free-flow calibration will underestimate tissue concentration. We develop mathematical models to better quantify the discrepancy between the calibration and tissue environment and experimentally verify our key predictions.

microelectrode biosensors are valuable experimental tools for accurate measurement of analytes in real time, both in vitro and in vivo (Dale et al. 2005). Biosensors have been used to measure neurotransmitters and neuromodulators including glutamate (Hu et al. 1994; Oldenziel et al. 2006; Tian et al. 2009), acetylcholine (Bruno et al. 2006; Zhang et al. 2010), adenosine triphosphate (Llaudet et al. 2005; Frenguelli et al. 2007; Gourine et al. 2008; Lalo et al. 2014; Lopatář et al. 2015; Wells et al. 2015), glucose (Lowry et al. 1998; Dash et al. 2013), adenosine, inosine, and hypoxanthine (Llaudet et al. 2003; Klyuch et al. 2012; Dale 2013; Van Gompel et al. 2014; Wall and Richardson 2015; Frenguelli and Wall 2016). Many microelectrode biosensors developed for brain tissue use oxidative enzymes followed by detection via fixed-potential amperometry. Such biosensors are typically formed of a platinum or carbon fiber core on which a conductive polymer matrix such as pyrrole or paraphenylene is electrochemically deposited. The thick layer biosensor designs considered in this paper are made when the enzyme is entrapped within the free volume of the polymer matrix (Llaudet et al. 2003) or bonded with the polymer matrix (Kotanen et al. 2014). The relevant feature of enzymatic biosensors for this study is that they break down and remove the quantity that they are measuring. To characterize the response of microelectrode biosensors and assist in their design, extensive mathematical and computational modeling has been used (Cambiaso et al. 1996; Lowry et al. 1998; Rinken and Tenno 2001), quantifying the influence of substrate and product inhibition (Simelevicius and Baronas 2010, 2011), geometry (Stikoniene et al. 2010), and enzyme kinetics (Ivanauskas et al. 2008; Simelevicius et al. 2012). However, interactions of the bulk properties of tissue with the biosensor, and how this scenario is distinct to calibration conditions, do not yet appear to have been fully considered.

Biosensors measure tissue concentrations of analytes by comparing the signal in tissue to that in calibration conditions. The biosensor is calibrated in a standard concentration of analyte, typically in free-flow conditions where the concentration of the analyte at the outer surface of the biosensor can be considered constant because any analyte broken down by the biosensor is rapidly replaced. Details of biosensor calibration are discussed in Frenguelli and Wall (2016). The free-flow calibration conditions differ substantially to those in tissue, where the analyte diffuses to reach the biosensor. Because the biosensor breaks down the analyte, it can be expected that a concentration gradient will be set up with a lower concentration near the biosensor surface than in the bulk tissue. The reduced volume fraction (porosity) and reduced diffusion coefficient due to obstructions (tortuosity) in tissue (Sykova and Nicholson 2008) will compound these effects.

Here we model this effect in free-flow, nontissue and tissue diffusive environments using an idealized description of a single-enzyme electrochemical biosensor. The model does indeed predict that in diffusive environments a density gradient is established with a reduced concentration near the biosensor: this central result is experimentally verified using single-enzyme biosensors (glucose and hypoxanthine) in agar blocks. The mathematical modeling provides a scaling factor that quantifies the discrepancy between free-flow and diffusive conditions. Although the scaling factor is strongly dependent on the properties of the particular analyte and tissue that is being investigated, it is apparent from its functional form that free-flow calibration will lead to significant underestimates of tissue concentrations.

## MATERIALS AND METHODS

### Concentrations, Fluxes, and Diffusion

#### Concentrations.

The concentrations considered are those in the volume fraction α where the compounds of interest (the analyte *A* or electroactive breakdown product *H*) can diffuse freely.

#### Fluxes.

In certain regions, such as within tissue, the extracellular space is highly tortuous, which has the effect of reducing the diffusion coefficient. For example, for a region denoted by b we use the diffusion permeability θ_b_ such that the effective diffusion coefficient is *D*_Ab_ = θ_b_*D*_A_, where *D*_A_ is the free diffusion coefficient for a particular compound (Sykova and Nicholson 2008). The flux uses the total concentration per unit volume (α*A*, for example) so for the analyte

(1) $$J_{\text{A}}=-\alpha_{\text{b}}\theta_{\text{b}}D_{\text{A}}\frac{\partial A}{\partial r}$$

would be the radial flux in the region b.

#### Boundary conditions.

At the interface between two regions, for example b and g, the concentrations (in the respective fractions of free space) at the boundary are matched as well as the fluxes across the boundary. For example for a boundary at, say, a radius *r*_2_ the continuity conditions would be

(2) $$A_{\text{b}}\left(r_{2}\right)=A_{\text{g}}\left(r_{2}\right)\;\text{and}\;\alpha_{\text{b}}\theta_{\text{b}}\frac{\partial A_{\text{b}}}{\partial r}|{}_{r_{2}}=\alpha_{g}\theta_{g}\frac{\partial A_{\text{g}}}{\partial r}|{}_{r_{2}}.$$

Note that the free diffusion coefficient *D*_A_ cancels from both sides in the flux condition.

#### Continuity at interfaces.

For cylindrical coordinates in which only the radial variable *r* is considered we have

(3) $$\nabla^{2}A=\frac{1}{r}\frac{\partial }{\partial r}\left(r\frac{\partial A}{\partial r}\right).$$

Neglecting the depth variable is a fair approximation for cylindrical boundary conditions and biosensors that are long relative to their radius, as is the case here.

### Description of Biosensor

**Fig. 1.**
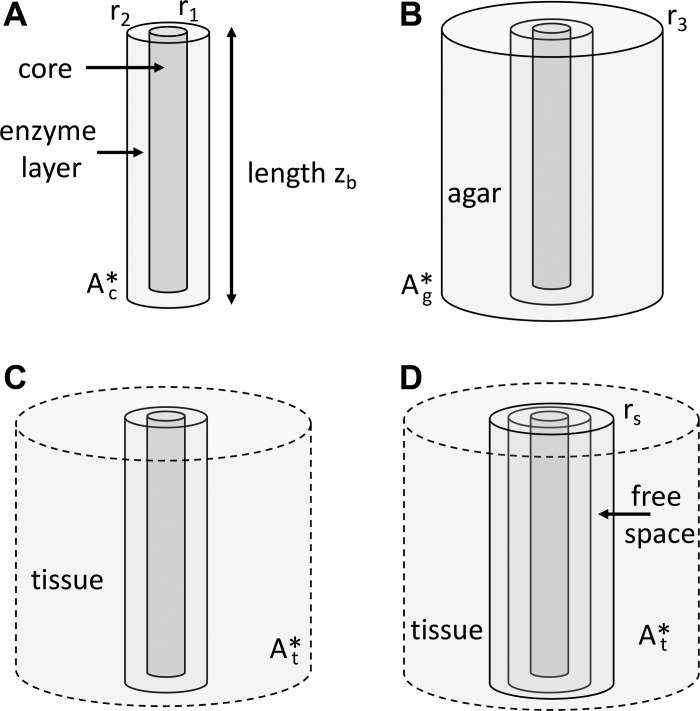
Schematic of the different model configurations. The biosensor electrode core has an outer radius of *r*_1_ and the biosensor enzyme layer extends from radii *r*_1_ to *r*_2_. The length of the biosensor is *z*_b_. *A*: calibration conditions. *B*: biosensor in agar, with the agar block extending out to a radius *r*_3_. *C*: biosensor in tissue, where the tissue is considered to be infinite in extent. Practically, this means extending out for a distance that is much greater than the tissue length constant ℓ_t_ (i.e., a few 100 μm) described in the related section in results. *D*: biosensor in tissue with a free-diffusion region caused by insertion damage extending from *r*_2_ to *r*_s_ beyond which the tissue begins.

The biosensor takes the form of a cylinder comprising an inner electrode core enveloped by an enzyme layer. The core radius is *r*_1_, the outer surface of the enzyme layer is at *r*_2_ and the length is *z*_b_ (Fig. 1*A*). Within the enzyme layer the analyte *A* is broken down into an electrically inactive product (which we ignore) and an electrically active product H_2_O_2_ hydrogen peroxide. It is this latter product that is measured at the electrode surface at radius *r*_1_. At the electrode we have

(4) $${\text{H}}_{2}{\text{O}}_{2}\to 2{\text{H}}^{+}+{\text{O}}_{2}+2e^{-}$$

so that two electrons are liberated for each H_2_O_2_ molecule. There are many characteristics used to quantify and compare biosensors (Baronas et al. 2009); however, here the current measured will be used. The current from the biosensor is equal to the charge on two electrons times the core area times the H_2_O_2_ flux per unit area at the core.

(5) $$I=2F\left(2\pi r_{1}z_{b}\right)J_{\text{H}}$$

where Faraday's constant *F* = 96,485 C/mol is the charge on a mole of electrons, 2π*r*_1_*z*_b_ is the surface area of the biosensor core and *J*_H_ is the H_2_O_2_ flux (note that contribution to the current coming from the surface area π*r*_1_^2^ of the end of the biosensor has been ignored this is a reasonable approximation for the length-radius ratio of the biosensors considered here).

### Model of Calibration Condition

For this condition the biosensor is placed in a free-flow environment with a constant concentration $A_{c}^{*}$ of the analyte (see Fig. 1*A*). Within the enzyme layer of the biosensor the analyte diffuses from the surface, with diffusion coefficient *D*_Ab_ = θ_b_*D*_A_ and is broken down at a constant rate *v*_b_ into H_2_O_2_, which diffuses within the enzyme layer with coefficient *D*_Hb_ = θ_b_*D*_H_. More complex enzyme kinetics have previously been considered (Baronas et al. 2009), but biosensors used in tissue are designed to operate within their linear regime so a linear-rate form is used here. The analyte and H_2_O_2_ concentrations *A*_b_(*r*) and *H*_b_(*r*) in the biosensor enzyme layer therefore obey

(6) $$\frac{\partial A_{\text{b}}}{\partial t}=D_{\text{Ab}}\nabla^{2}A_{\text{b}}-v_{\text{b}}\;A_{\text{b}}$$

(7) $$\frac{\partial H_{\text{b}}}{\partial t}=D_{\text{Hb}}\nabla^{2}H_{\text{b}}+v_{\text{b}}\;A_{\text{b}}.$$

At the core radius *r*_1_ there is zero flux of the analyte and H_2_O_2_ is rapidly removed and so has zero concentration

(8) $$\frac{\partial A_{\text{b}}}{\partial t}|{}_{r_{1}}=0\;\text{and}\;H_{\text{b}}\left(r_{1}\right)=0.$$

At the surface of the biosensor at *r*_2_ there is continuity of analyte concentration and zero concentration of the breakdown product, because it is rapidly washed away in the free-flow environment. Hence, the following boundary conditions hold at the outer surface of the biosensor enzyme layer

(9) $$A_{\text{b}}\left(r_{2}\right)=A_{\text{c}}^{*}\;\text{and}\;H_{\text{b}}\left(r_{2}\right)=0.$$

Finally, the current measured on the biosensor is proportional to minus the radial flux H_2_O_2_ at the core (radius *r*_1_)

(10) $$I=\left(2F\right)\left(2\pi r_{1}z_{\text{b}}\right)\alpha_{\text{b}}D_{\text{Hb}}\frac{\partial H_{\text{b}}}{\partial r}|{}_{r_{1}}$$

for a biosensor of length *z*_b_ (end effects are ignored in this model).

### Model of Diffusive Conditions in Agar

For this condition we consider the biosensor embedded within a long cylindrical block of agar that is immersed in a bathing medium with concentration $A_{g}^{*}$ at the agar outer surface (see Fig. 1*B*). The motion of the analyte *A*_g_ and the breakdown product *H*_g_ within the agar are diffusive with diffusion coefficients *D*_Ag_ and *D*_Hg_, respectively. The free-volume fraction is α_g_ and the diffusion permeability is θ_g_. The equations of motion are therefore

(11) $$\frac{\partial A_{g}}{\partial t}=D_{\text{Ag}}\nabla^{2}A_{\text{g}}$$

(12) $$\frac{\partial H_{g}}{\partial t}=D_{\text{Hg}}\nabla^{2}H_{\text{g}}.$$

Within the biosensor enzyme layer the analyte and H_2_O_2_ obey *Eqs. 6* and *7* with boundary conditions at the core radius *r*_1_ given by *Eq. 8* and the biosensor current by *Eq. 10*. However, at the biosensor surface we require continuity so that

(13) $$A_{\text{b}}\left(r_{2}\right)=A_{\text{g}}\left(r_{2}\right)\;\text{ and}\;\;\alpha_{\text{b}}\theta_{\text{b}}\frac{\partial A_{\text{b}}}{\partial r}|{}_{r_{2}}=\alpha_{\text{g}}\theta_{\text{g}}\frac{\partial A_{\text{g}}}{\partial r}|{}_{r_{2}}$$

with identical continuity conditions for *H*_b_ and *H*_g_ at *r*_2_. Similarly at the surface of the agar we have the conditions

(14) $$A_{\text{g}}\left(r_{3}\right)=A_{\text{g}}^{*}\;\text{and}\;H_{\text{g}}\left(r_{3}\right)=0$$

where it is assumed that there is no breakdown product *H* in the bathing medium because any that diffuses out is rapidly washed away.

### Coarse-Grained Description of Tissue

Macroscopic models of complex media involve spatial averaging over an appropriate volume such that a homogeneous description becomes valid. For neural tissue this means that concentrations of analyte represent averages over length scales of ∼10 μm (Hrabe et al. 2004; Sykova and Nicholson 2008). There are two key factors that affect the diffusive motion of analyte through tissue. The first is the porosity of the tissue that leads to a reduced free-volume fraction α_t_ in tissue through which the analyte can diffuse. If the analyte cannot enter cells then the reduced volume fraction is typically α_t_ ≃ 0.2 in brain tissue (Sykova and Nicholson 2008) corresponding to diffusion in the extracellular space only. If the analyte can enter and leave cells, this generally leads to a more complex model that is beyond the scope of the current paper. The second factor that affects the diffusive motion of the analyte is the tortuosity λ of the tissue resulting from the complex microscopic structure around which the analyte diffuses. This can be modeled by using a diffusion coefficient *D*_At_ = θ_t_*D*_A_ for the analyte, that is reduced from that in a free solution *D*_A_ by a permeability factor θ_t_ = 1/λ^2^. For brain tissue the tortuosity is quoted as λ = 1.6 (Sykova and Nicholson 2008) giving θ_t_ ≃ 0.4. We assume that the electroactive breakdown product *H* is very rapidly removed from tissue (as is the case for hydrogen peroxide). Its concentration is therefore considered to be zero throughout the tissue.

### Model of Biosensor in Tissue

The tissue is considered to have some steady-state concentration of the analyte $A_{t}^{*}$ far away from the biosensor (see Fig. 1*C*). This is maintained by an equilibrium between some unspecified release mechanism and a tissue removal mechanism at rate *v*_t_. Mathematically, this can be captured in the following description

(15) $$\frac{\partial A_{\text{t}}}{\partial t}=D_{\text{At}}\nabla^{2}A_{\text{t}}+v_{\text{t}}\left(A_{\text{t}}^{*}-A_{\text{t}}\right)$$

with the behavior of the analyte and breakdown product in the biosensor enzyme layer obeying the substrate *Eqs. 6* and *7*. Note that there is no tissue equation for *H* because it is considered to be rapidly removed from tissue and so the boundary condition *H*_b_(*r*_2_) = 0 also applies for the tissue condition. The boundary conditions for the analyte at the interface are now

(16) $$A_{\text{b}}\left(r_{2}\right)=A_{\text{t}}\left(r_{2}\right)\;\text{ and}\;\;\alpha_{\text{b}}\theta_{\text{b}}\frac{\partial A_{\text{b}}}{\partial r}|{}_{r_{2}}=\alpha_{\text{t}}\theta_{\text{t}}\frac{\partial A_{\text{t}}}{\partial r}|{}_{r_{2}}$$

where the last equality ensures that the analyte fluxes across the biosensor interface are matched.

### Model of Biosensor in Tissue with Free Space

Insertion of the biosensor can sometimes damage surrounding tissue. This aspect was included in the modeling as a free diffusion space in a region from the biosensor surface at a radius *r*_2_ to a radius *r*_s_ beyond which the tissue extends (see Fig. 1*D*). The dynamics within the biosensor and the tissue follow *Eqs. 6*, *7*, and *15* as before. Within the free-diffusion space the dynamics are

(17) $$\frac{\partial A_{\text{s}}}{\partial t}=D_{\text{A}}\nabla^{2}A_{\text{s}}\;\text{ and}\;\;\frac{\partial H_{\text{s}}}{\partial t}=D_{\text{H}}\nabla^{2}H_{\text{s}}.$$

The boundary conditions at the biosensor surface are

(18) $$A_{\text{b}}\left(r_{2}\right)=A_{\text{s}}\left(r_{2}\right)\;\text{ and}\;\;\alpha_{\text{b}}\theta_{\text{b}}\frac{\partial A_{\text{b}}}{\partial r}|{}_{r_{2}}=\frac{\partial A_{\text{s}}}{\partial r}|{}_{r_{2}}$$

and similarly for the *H* variable. At the interface between the free space and tissue we have

(19) $$A_{\text{s}}\left(r_{s}\right)=A_{t}\left(r_{s}\right)\;\;\text{ and}\;\;\;\frac{\partial A_{\text{s}}}{\partial r}|{}_{r_{\text{s}}}=\alpha_{\text{t}}\theta_{\text{t}}\frac{\partial A_{\text{t}}}{\partial r}|{}_{r_{2}}$$

with *H*_t_(*r*_s_) = 0 providing the boundary condition for *H*.

### Analytical and Numerical Solutions

#### Analytical solutions.

The models considered here have radial symmetry and so the steady-state equations typically take the form

(20) $$\nabla^{2}A=0\;\;\text{ or}\;\;\ell^{2}\nabla^{2}A=A$$

with similar equations for *H*. The first equation is satisfied by a constant plus a logarithm log(*r*) multiplied by a constant. The second equation is satisfied by a linear combination of the zero-order modified Bessel functions *I*_0_(*r*/ℓ) and *K*_0_(*r*/ℓ). The solutions for each of the cases considered here are provided in the appendix.

#### Numerical solutions.

We sought analytical solutions for the coupled differential equations describing the steady state and numerical solutions for the partial differential equations describing the time-dependent concentration profiles. Analytical solutions for the steady state are derived in the appendix. For the numerical solution of the partial differential equations, the system was discretized in time and space and integrated forward in time using a second-order or fourth-order Runge-Kutta scheme.

### Experimental Methods

All microelectrode biosensors were obtained from Sarissa Biomedical (Coventry, UK). Microelectrode biosensors consist of an enzymatic biolayer on top of a permselectivity layer around a Pt/Ir wire (diameter: 50 μm), which has a length of 500 μm. A block of agar (0.6–0.9 g in 50 ml, ∼2 mm^3^) was held submerged in a recording bath and perfused (6 ml/min) with recording saline composed of the following (in mM): 127 NaCl, 1.9 KCl, 1 MgCl_2_, 2 CaCl_2_, 1.2 KH_2_PO_4_, and 26 NaHCO_3_ (pH 7.4 when bubbled with 95% O_2_-5% CO_2_, 300 mosM) at 32°C. The block of agar sat on a suspended grid so was perfused from above and below. Biosensors were positioned above the agar block, in the bathing medium, and polarized. The agar and biosensors were perfused with the analyte of interest for 20–30 min to allow equilibration. Biosensors were manually inserted (in <1 s) into the agar block so that the sensing area was completely embedded. Biosensor signals were acquired at 1 KHz with a Micro 1401 interface using Spike 2 (Vs 6.14) software (Cambridge Electronics Design, Cambridge, UK). Glucose biosensors have entrapped glucose oxidase in an enzyme layer, which oxidizes glucose to d-glucono-1,5-lactone + H_2_O_2_ (Updike and Hicks 1967; Frayling et al. 2011). Hypoxanthine biosensors have entrapped xanthine oxidase in an enzyme layer, which oxidizes hypoxanthine to xanthine + H_2_O_2_ and also xanthine + O_2_ to uric acid + H_2_O_2_. Experiments typically used 50 μM glucose or 10 μM hypoxanthine. Values are quoted as the means ± SD based on *n* trials.

### Parameter Choice

The parameters used are summarized in Table 1. The approach was to use a generic model of a single-enzyme biosensor in which the analyte has relatively simple dynamics in tissue. Reasonable values for the quantities were chosen in respect to specific biosensors, such as hypoxanthine or glucose biosensors.

**Table 1.** Parameters used in the paper, unless otherwise stated

Parameter	Value	Description
*r*_1_	25 μm	Biosensor core radius^1^
*r*_2_	50 μm	Biosensor outer radius^1^
*r*_3_	150 μm	Agar block outer radius (for Fig 3)
*z*_b_	500 μm	Biosensor length^1^
*D*_A_	860 μm^2^/s	Glucose free diffusion coefficient^2^
*D*_H_	1,700 μm^2^/s	H_2_O_2_ free diffusion coefficient^2^
*v*_b_	100/s	Biosensor reaction rate*
α_b_	0.4	Biosensor free-volume fraction^3^
θ_b_	1.0	Biosensor diffusion permeability^1^
α_g_	1.0	Agar free-volume fraction^4^
θ_g_	1.0	Agar diffusion permeability^5^
*v*_t_	0.1/s	Tissue reaction rate*
α_t_	0.2	Tissue free-volume fraction^6^
θ_t_	0.4	Tissue diffusion permeability^6^

1 Sarissa Biomedical;

2 van Stroe-Biezen et al. 1993;

3 Hallik et al. 2007;

4 McCabe 1972;

5 Nicholson et al. 1979;

6 Sykova and Nicholson 2008.

* Generic parameters.

#### Biosensor dimensions.

Several sizes of biosensors are available, the most common size provided by Sarissa Biomedical have a core of radius *r*_1_ = 25 μm. The outer surface of the enzyme layer, although more variable, was typically *r*_2_ = 50 μm. The length of the core was *z*_b_ = 500 μm.

#### Volume fractions and tortuosity.

The volume fraction α_b_ and tortuosity θ_b_ are rarely if ever specified in publications on biosensor design. The free volume fraction in the polypyrrole matrix is difficult to determine, as it is known to depend on deposition conditions, doping agents, inert additives, and thickness (Garcia-Belmonte 2003). A comparison of the free volume of polypyrrole with different dopant-ions measured with nitrogen gas (Hallik et al. 2007) has volume fractions between 0.26 and 0.56, but nitrogen is much smaller than either hydrogen peroxide, glucose, or hypoxanthine and so may overestimate the relevant free volume. Here the value α_b_ = 0.4 is chosen. The diffusion coefficient in the polymer matrix has been quoted (Sarissa Biomedical) as being similar to that for free diffusion and so θ_b_ = 1.0 is used. For the agar we assume little excluded volume α_b_ = 1.0 and low tortuosity and so that θ_g_ = 1.0 (McCabe 1972; Nicholson et al. 1979). The corresponding quantities α_t_ and θ_t_ for tissue are described above.

#### Free diffusion coefficients.

The free diffusion coefficients for glucose and hydrogen peroxide at 32°C are 860 and 1,700 μm^2^/s (van Stroe-Biezen et al. 1993).

#### Biosensor breakdown rates.

The model is meant to be generic and describes diffusion rather than enzyme limited biosensors, so a representative and relatively rapid breakdown rate of analyte in the biosensor enzyme layer of *v*_b_ = 100/s is chosen. Such a rapid rate is seen for specific example in the hypoxanthine biosensor, which is constructed with 10 μl containing the pyrrole monomer and 5 U xanthine oxidase (Llaudet et al. 2003) of which ∼8% is immobilized (Coche-Guerente 1995). Assuming the enzyme is uniformly distributed, the kinetics are not significantly affected by entrapment in the polymer matrix and using average velocity as a maximum velocity provides an estimated removal rate of *v*_b_ = 178/s (Monda and Mitra 1994).

#### Tissue breakdown rates.

Similarly for the tissue breakdown rate a generic value of *v*_t_ = 0.1/s is chosen. This obviously depends on the analyte in question, but generally it is relatively slow compared with that in the biosensor enzyme layer. This is reasonable for hypoxanthine, for example. In homogenate of the rat cerebrum and cerebellum, xanthine oxidase activity was found to be 19.5 mU/g of tissue at 30°C (Hashimoto 1974). Assuming hypoxanthine clearance is due to xanthine oxidase and this activity is uniformly distributed in the brain suggests a clearance rate *v*_t_ = 40 × 10^−3^/s (Coche-Guerente 1995). Considering glucose as another example, its metabolism in the brain is a complex process involving multiple metabolic pathways and is coupled with neuronal activity (Bèlanger et al. 2011). An estimate can be obtained from studies using radiolabeled analogs (Berti et al. 2013). Assuming labeled analog behavior is the same as glucose, the phosphorylation rate in grey matter is ∼1.2 × 10^−3^/s for human or ∼0.9 × 10^−3^/s for rats (Reivich et al. 1985). Glucose is transported though the blood-brain-barrier at a similarly slow rate, ∼0.2 × 10^−3^/s (Clarke and Sokoloff 1999).

## RESULTS

First, the steady-state concentration profile for the idealized biosensor is modeled in the free-flow calibration condition. We then examine the response when the biosensor is placed in an environment where the analyte reaches the sensor by diffusion. These model results are then tested experimentally in a condition where the biosensor is inserted into a block of agar in which the analyte reaches the biosensor through diffusion. This verifies the key finding that in diffusive environments the biosensor measures a smaller concentration than in free-flow conditions due to the density gradient being set up. The implication for this effect in tissue is then modeled using a general model for an analyte being generated and cleared in tissue.

### Model of Free-Flow Calibration

**Fig. 2.**
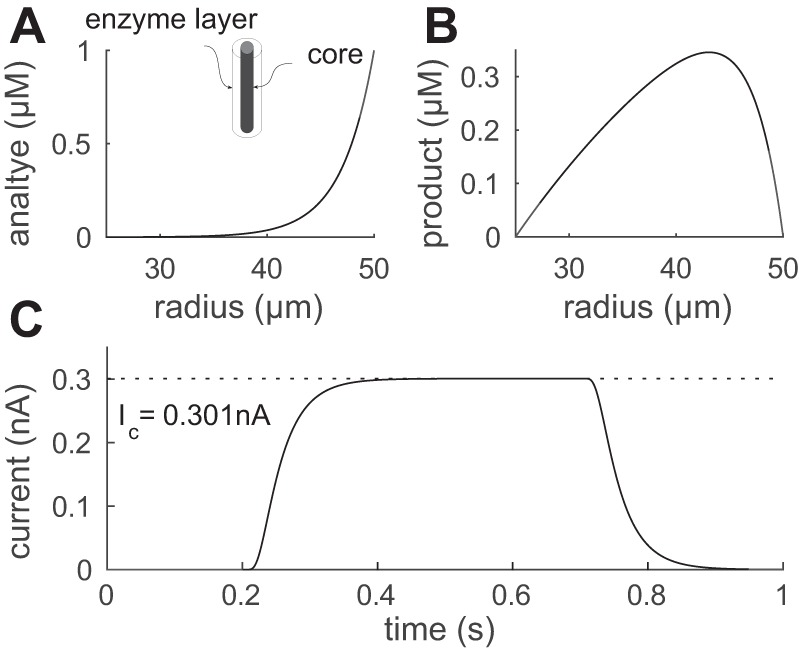
Model: calibration condition. *Inset*: experimental configuration. The enzyme layer is from a radii 25 to 50 μm. *A*: the steady-state analyte concentration sharply decreases from the surface for this diffusion-limited biosensor, so peak breakdown H_2_O_2_ production is at the biosensor surface. *B*: corresponding concentration profile for H_2_O_2_. *C*: the dynamics of the biosensor current demonstrating the rapid (<1 s) responsiveness. The bath concentration of analyte was $A_{c}^{*}$ = 1 μM with other parameters given in Table 1.

A biosensor is typically calibrated by placing it in a free-flow environment in which the analyte *A* has a fixed concentration $A_{c}^{*}$ at its outer surface (Frenguelli and Wall 2016). As the analyte is absorbed into the biosensor enzyme layer, it diffuses until it is broken down by the enzyme at a rate *v*_b_ into a certain number of molecules of the electrically active product H_2_O_2_, the concentration of which we denote by *H*. Mathematically this can be described by *Eqs. 6* and *7* with boundary conditions given by *Eqs. 8* and *9*. Biophysically, these conditions assume that the analyte *A* can freely cross the outer boundary of the biosensor enzyme layer but cannot diffuse into the solid electrode core, whereas for the breakdown product *H* we assume that the concentration is zero outside the biosensor due to the free-flow condition and that *H* is broken down when coming into contact with the biosensor core. It is therefore the flux of *H* into the biosensor core that is proportional to the measured signal. An example, for steady-state concentrations, is given in Fig. 2*A* using parameters from Table 1. For the parameter values used the biosensor rapidly metabolizes the analyte such that very little reaches the biosensor core before being broken down. The resulting breakdown product either diffuses out of the biosensor and is lost or diffuses to the core and is electrolyzed and measured as a signal. Mathematically, the signal measured takes the form

(21) $$I_{\text{c}}=2Fz_{\text{b}}\frac{2\pi \alpha_{\text{b}}D_{\text{Ab}}}{\log\left(r_{2}/r_{1}\right)}\left(A_{b}\left(r_{2}\right)-A_{b}\left(r_{1}\right)\right)$$

where *A*_b_(*r*) is the analyte concentration in the biosensor enzyme layer at a radius *r* (see the appendix). Interestingly, this quantity does not depend on the diffusion coefficient of the breakdown product *H* in the biosensor. A characteristic diffusion length ℓ_b_ can be derived for the analyte in the biosensor, which is given by ℓ_b_^2^ = *D*_Ab_/*v*_b_. This is the typical distance that a molecule of analyte will diffuse into the biosensor enzyme layer before being broken down and will be relatively small for a diffusion limited biosensor e.g., ℓ_b_ = 1.9 μm for parameters used here. Note that if ℓ_b_ ≪ (*r*_2_ − *r*_1_), then there is little chance that the analyte reaches the core before break down, so that *A*_b_(*r*_1_) ≪ *A*_b_(*r*_2_) and then the current simplifies to

(22) $$I_{\text{c}}≃2Fz_{\text{b}}\frac{2\pi \alpha_{\text{b}}D_{\text{Ab}}}{\log\left(r_{2}/r_{1}\right)}A_{\text{c}}^{*}$$

where the boundary condition *A*_b_(*r*_2_) = $A_{c}^{*}$ has been used. This is a particularly simple form in that it does not depend on the diffusion coefficient of *H* or the breakdown rate *v*_b_. These free-flow calibration results (Fig. 2) will now be compared with diffusive environments (agar and tissue).

### Model of a Biosensor in Agar

**Fig. 3.**
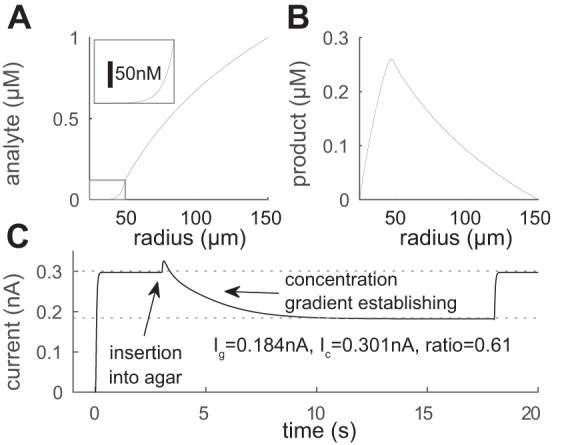
Model: biosensor in cylindrical agar block (see schematic *inset*) with the biosensor enzyme layer extending from radii 25 to 50 μm and the agar block from radii 50 to 150 μm. *A*: the steady-state analyte concentration exhibits a density gradient from the agar surface that is induced by breakdown within the biosensor enzyme layer. *Inset*: detail within the enzyme layer itself. *B*: corresponding concentration of H_2_O_2_. Note that H_2_O_2_ is lost through diffusion into the agar and then washed away. *C*: dynamics of the current response for the biosensor inserted into the bath (calibration condition) from 0 to 3 s into the agar from 3 to 18 s during which the density gradient builds up and finally back into the bath from 18 s onwards. The increase at the point of insertion into the agar is due to the transient increase in local H_2_O_2_ concentration, which was often seen in experiment (see Fig. 4).

Agar provides a diffusive environment similar to tissue (though there is no excluded volume) but without the added complexities of endogenous analyte dynamics. We now consider the case of a long biosensor embedded in a cylindrical block of agar with concentration $A_{g}^{*}$ at the surface of the agar (Fig. 3, *inset*). The steady-state current at the biosensor for this configuration is straightforward to derive and is provided in the appendix. Because the biosensor acts as a sink and a diffusion gradient is set up, the concentration of the analyte at the biosensor surface is reduced by a factor *c*_g_ so that *A*(*r*_2_) = $c_{\text{g}}A_{g}^{*}$ where *c*_g_ is defined (57). Provided the length scale is small relative to the size of the biosensor core, so that ℓ_b_ ≪ *r*_1_, we have

(23) $$c_{g}≃\frac{\ell_{\text{b}}}{\ell_{\text{b}}+\frac{\alpha_{\text{b}}\theta_{\text{b}}}{\alpha_{\text{g}}\theta_{\text{g}}}r_{2}\,\;\log\left(r_{3}/r_{2}\right)}.$$

For the parameters used in Fig. 3, this reduction is substantial (88%). However, the current itself is not so significantly attenuated because some of the electroactive breakdown product H_2_O_2_ generated by the biosensor and initially lost into the agar diffuses back to the biosensor matrix. The form of the current can be written

(24) $$I_{\text{g}}=2Fz_{\text{b}}\frac{2\pi \alpha_{\text{b}}D_{\text{Ab}}}{\log\left(r_{2}/r_{1}\right)}\left(A_{\text{g}}^{*}-A_{1}\right)\kappa_{\text{b}},$$

for a biosensor of length *z*_b_, where the constant is

(25) $$\kappa_{b}=\frac{1}{1+\frac{\alpha_{\text{b}}\theta_{\text{b}}}{\alpha_{\text{g}}\theta_{\text{g}}}\frac{\log\left(r_{3}/r_{2}\right)}{\log\left(r_{2}/r_{1}\right)}}.$$

The inferred concentration of analyte in agar, obtained by comparison of the biosensor current in agar with that in calibration condition, underestimates the true concentration

(26) $$A_{\text{inferred}}^{*}=A_{\text{g}}^{*}\frac{\left(1-c_{\text{g}}X\left(r_{1}\right)\right)}{\left(1-X\left(r_{1}\right)\right)}\kappa_{b}.$$

For diffusion-limited biosensors the quantity *X*(*r*_1_) ≪ 1 and so a good approximation is a reduction by a factor κ_b_

(27) $$A_{\text{true}}^{*}≃A_{\text{inferred}}^{*}\left(1+\frac{\alpha_{\text{b}}\theta_{\text{b}}}{\alpha_{\text{g}}\theta_{\text{g}}}\frac{\log\left(r_{3}/r_{2}\right)}{\log\left(r_{2}/r_{1}\right)}\right).$$

Hence, even if the tortuosities and the free volume fractions are the same in the enzyme layer and surrounding agar block and *r*_2_/*r*_1_ = *r*_3_/*r*_2_, the current-equivalent concentration predicted would be 50% lower. This clearly indicates a mismatch between the free-flow calibration conditions and the diffusive experimental environment (in agar or tissue) that is likely to result in underestimation of analyte concentrations.

### Experimental Verification of Calibration Mismatch

**Fig. 4.**
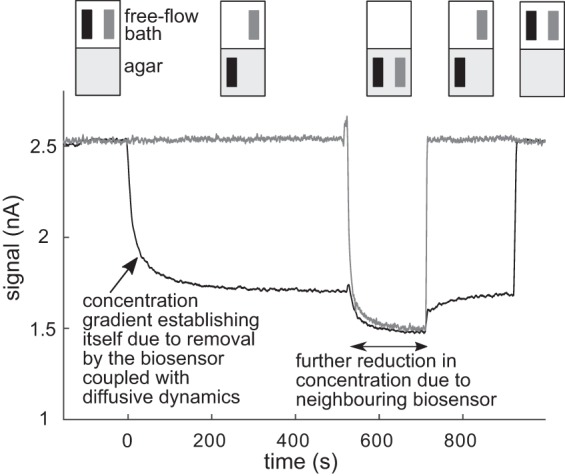
Experiment: diffusive transport in agar markedly reduces the biosensor current. Two glucose biosensors, with almost identical sensitivities, were moved in and out of an agar block. *Top*: configurations of the 2 biosensors (black and grey) either above the agar block (free-flow conditions) or inserted into the agar. *Bottom*: the respective, superimposed current traces from *top*. Initially both biosensors were held in free-flow calibration conditions in the presence of 50 μM glucose (current of ∼2.5 nA). The first biosensor (black) was then fully inserted into the agar block. The current recorded dropped to ∼ 1.75/2.5 = 70% of its calibration value due to the establishment of the diffusion gradient. To verify the presence of the concentration gradient the second biosensor was then inserted close to the first. An initial rise due to a transient and localized increase in H_2_O_2_ can be seen, as predicted by the model (Fig. 3). The second biosensor steady-state signal was lower than that of the first biosensor previously; however, the first biosensor signal also dropped to the same lower value. These results are what would be expected if each biosensor established a density gradient of analyte and that these gradients superpose. On removal of the second biosensor from the agar the first biosensor recovered to the earlier steady current of ∼1.75 nA. Both biosensor signals returned to their calibration values when removed from the agar.

Because it is technically difficult to cut an agar block into a near perfect cylinder, we performed an experiment with a slightly altered geometry. A large rectangular block of agar was immersed in a free-flowing bath with a constant concentration of analyte. Compared with the free-flow conditions, there was a substantial and rapid drop in the signal when a biosensor was inserted into the agar for both hypoxanthine 52.4% (22.6%, *n* = 5) and glucose 43.2% (8.0%, *n* = 5) biosensors. A typical experiment is illustrated in Fig. 4. Here two glucose biosensors, with similar sensitivity, were initially held within the flow (equivalent to calibration conditions) with 50 μM glucose and both registered a current of ∼2.5 nA (average of first 100 s). The first biosensor was then fully inserted into the agar. The signal then decreased with a slow decay rate (∼600 s) and reached a new value near 1.75 nA. To test that the decrease is due to a density gradient being set up, the second biosensor was then introduced into the agar near the first biosensor. This resulted in a further decrease in the current on the first biosensor, with both biosensors reaching a new, lower steady-state current of ∼1.5 nA (a similar result was obtained with hypoxanthine biosensors, with the decrease depending on both the distance between them and difference in the angle of insertion). On removal of the second biosensor the signal on the first biosensor recovered to its previous value of 1.75 nA. Note that when the first biosensor was removed and placed back into calibration conditions the recovery to the calibration current was very rapid (<5 s). This makes it clear that the slow dynamics seen during the insertion into the agar are not inherent to the biosensor but rather to the setting up of the diffusion gradient, as predicted by the model in the previous section. When a single biosensor is inserted together with a null sensor (one lacking an enzyme layer), there was little or no effect on the biosensor current; however, a small increase in the null-sensor current (∼50–100 pA) was seen as would be expected from H_2_O_2_ diffusive overspill from analyte breakdown in the active biosensor.

### Model of a Biosensor in Tissue

Although the analyte diffuses to reach the biosensor in both agar and tissue, there are key differences between the conditions. In tissue there is a significantly reduced volume fraction α_t_ and the tortuosity is greater resulting in a smaller diffusion constant *D*_At_. Additionally the analyte, rather than being externally applied, is generated within the tissue itself. We model the latter property by considering a balance between the release rate and breakdown rate *v*_t_, resulting in a steady-state concentration of $A_{t}^{*}$ that is homogeneous throughout the tissue (far away from the biosensor). On the insertion of the biosensor, the analyte will begin to diffuse into the enzyme layer and be broken down, resulting in a density gradient being set up in the tissue around the biosensor. An analysis of the system gives a length ℓ_t_, defined through ℓ_t_^*^ = *D*_At_/*v*_t_, which gives a scale for the range of influence of the biosensor in tissue. For the parameters used this length was 59 μm. The tissue case can be modeled by using the biosensor equations for *A* and *H* within the enzyme layer coupled to equation (15) for *A* in the tissue. The boundary condition in this case is that far (*r* ≫ ℓ_t_) from the biosensor that the tissue concentration approaches $A_{\mathrm{t.}}^{*}$ There are a number of subsidiary conditions we could use for H_2_O_2_ in tissue. In this section we firstly consider that the breakdown product *H* is instantly removed from tissue. Under these circumstances the profile of the analyte *A*_b_ and breakdown product *H*_b_ in the biosensor is functionally the same as the calibration case (although with different amplitudes): all that remains is to find the reduction in analyte concentration at the biosensor surface. Writing this in the form *A*(*r*_2_) = ${c_{t}A_{t}^{*}}_{}^{}$ the constant, as shown in the appendix, can be written

(28) $$c_{\text{t}}={\left(1-\frac{\alpha_{\text{b}}D_{\text{Ab}}}{\alpha_{\text{t}}D_{\text{At}}}\frac{{X\prime }^{}\left(r_{2}\right)}{{Y\prime }^{}\left(r_{2}\right)}\right)}^{-1}$$

where *X*(*r*) and *Y*(*r*) and their derivatives are also defined in the appendix. Note that this quantity is strictly positive as the gradient of *Y*(*r*) is negative, cancelling out the apparent minus sign. Because of the boundary conditions, the biosensor current takes the same form as in *Eq. 21* but with (*A*_2_ − *A*_1_) reduced by the factor *c*_t_ so that

(29) $$A_{\text{true}}^{*}=A_{\text{inferred}}^{*}\left(1-\frac{\alpha_{\text{b}}D_{\text{Ab}}}{\alpha_{\text{t}}D_{\text{At}}}\frac{{X\prime }^{}\left(r_{2}\right)}{{Y\prime }^{}\left(r_{2}\right)}\right).$$

**Fig. 5.**
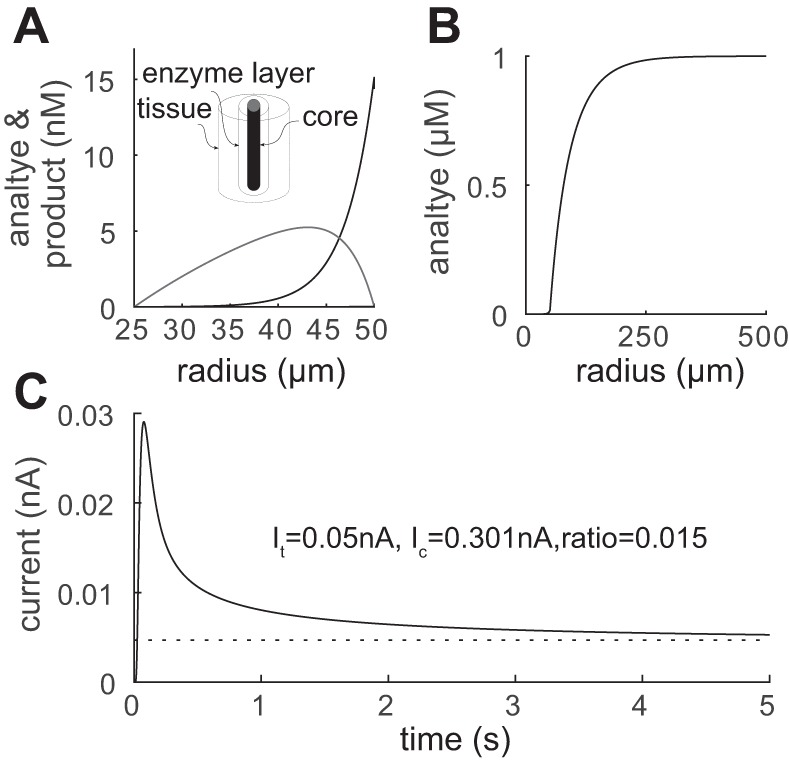
Model: biosensor in tissue. *A*: concentration of analyte and H_2_O_2_ in the biosensor enzyme layer. Note the same forms as Fig. 2. *B*: distribution of the analyte in tissue. A sharp decrease in density around the biosensor from the bulk value of 1 μM is apparent. *C*: time course of the biosensor current. The current is initially high but then decreases as the analyte around the biosensor is broken down and the density gradient is set up. In this particular example the biosensor measures a steady-state current equivalent to only ∼1.5% of the bulk tissue concentration. This mismatch is much greater than the case for agar, largely due to the instantaneous removal of H_2_O_2_ in this model of tissue.

Because any H_2_O_2_ leaving the biosensor is rapidly broken down once it enters the tissue, the mismatch between calibration and tissue measurements is substantial in this model: for the example in Fig. 5 the tissue concentration would be measured at only 1.5% of its value in tissue far away from the biosensor.

### Effect of Space Around Biosensor in Tissue

**Fig. 6.**
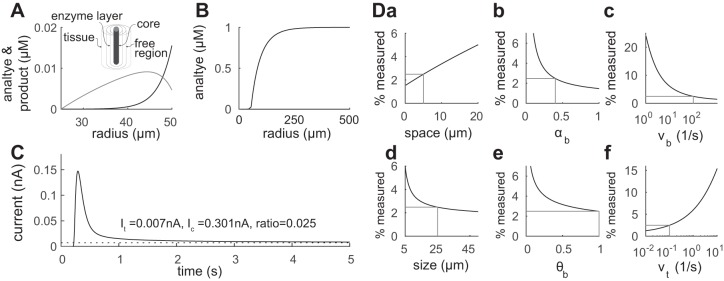
Model: biosensor surrounded by a free-diffusion layer in tissue. *A*: concentrations of analyte and H_2_O_2_ in the biosensor enzyme layer. Here the functional forms of the concentration profiles differ from Fig. 2 due to the boundary conditions for *H* on the biosensor surface. *B*: the distributions of the analyte in tissue. *C*: time course of the biosensor current. In this example the biosensor measures a steady-state signal equivalent to only ∼ 2.5% of the concentration in tissue far from the biosensor. This is nevertheless less of a mismatch than for the model without a free space around the biosensor, in Fig 5. *D*: examination of the calibration mismatch as key parameters are varied; *a*: size of the free space *r*_s_ − *r*_2_; *b*: porosity α_b_ of the biosensor enzyme layer; *c*: breakdown rate *v*_b_ in biosensor; *d*: the radius *r*_2_ of the biosensor (with equal core radius and enzyme layer thickness); *e*: diffusion permeability θ_b_ in the biosensor enzyme layer; and *f*: breakdown rate *v*_t_ in tissue. The parameters used in *A–C* are shown in grey.

We now consider the effect of a thin region around the biosensor that allows free diffusion. As well as being of biophysical relevance, it can be caused by the insertion of the biosensor into the tissue the analysis also serves to demonstrate how sensitive calibration-correction factors are to details of the biosensor-tissue interactions. The free space considered extends from the biosensor surface at *r*_2_ to a radius *r*_s,_ and then for radii greater than *r*_s_, the tissue conditions are the same as in the previous case. The concentration mismatch now takes the form

(30) $$A_{\text{inferred}}=A_{\text{c}}^{*}\frac{I_{\text{tis}}}{I_{\text{cal}}}=\kappa_{b}\gamma_{b}A_{\text{t}}^{*}$$

where κ_b_ and γ_b_ have a fairly complex dependency on the parameters given by *Eqs. 75* and *79* of the appendix. The effect of adding a free-diffusion space of *r*_s_ = *r*_2_ + 5 μm is shown in Fig. 6. As can be seen, even a small region of free diffusion can have a significant effect on the concentration. For the parameters used here, the percentage of the true concentration measured rises to 2.5% from the 1.5% seen for the previous case of no free-diffusion region (*r*_s_ = *r*_2_). This effect underlies that the calibration mismatch for tissue conditions is a complex quantity that has a strong dependence on the diffusion of H_2_O_2_ into tissue and back. To examine the functional dependency of the calibration mismatch we varied a number of key parameters:

#### Free-diffusion space.

The effect of increasing *r*_s_ led to a broadly linear (Fig. 6*Da*) improvement in the biosensor measure, over a range of *r*_s_ − *r*_2_ up to 20 μm.

#### Biosensor free volume and permeability.

The accuracy of the biosensor measurement increases markedly with decreasing free-volume fraction α_b_ or diffusion permeability θ_b_ of the biosensor enzyme layer (Fig. 6, *Db* and *De*).

#### Breakdown rates.

The calibration mismatch becomes worse with decreasing biosensor reaction rate *v*_b_ (Fig. 6*Dc*). This can be expected as the biosensor is destroying less of the analyte and so the density gradient is reduced. However, in constructing a biosensor it is not desirable to have too low a reaction rate as this leads to a poor signal-to-noise ratio, similar to the reduced size of the biosensor. For a faster reaction rate in tissue *v*_t_, the calibration mismatch is less severe (Fig. 6*Df*), which can be understood as fixing $A_{t}^{*}$ meaning that the relative replenishment rate of analyte is higher too.

#### Biosensor size.

Biosensors are available in various sizes, here we consider the core radius to be equal to the thickness of the enzyme layer and consider a range of sizes from 10 μm (with 5-μm core and enzyme layer) to 100 μm (with 50-μm core and enzyme layer). The calibration mismatch is less for smaller biosensors (Fig. 6*Dd*) due primarily to the smaller enzyme layer. However, the typical distance the analyte diffuses through the enzyme layer before being oxidized is ℓ_b_ = 1.9 μm, so even for the smallest size considered, most of the electroactive product is lost to the tissue.

#### Diffusion constants.

For the model assumptions made in this paper, the steady-state biosensor current is unaffected by the diffusion coefficient of H_2_O_2_ and the calibration mismatch is not substantially altered by the diffusion coefficient of the analyte. In summary, the sensitivity analysis demonstrates that conventional calibration will substantially underestimate the concentration of analyte in tissue for a range of parameters relevant to current microelectrode-biosensor usage.

## DISCUSSION

Microelectrode biosensors provide good spatiotemporal resolution for measurements of a range of physiologically relevant substances in vitro and in vivo (Dale et al. 2005) and are widepread in their use. Here we presented models of a generic single-enzyme biosensor under calibration conditions in agar and in tissue. We clearly demonstrate a discrepancy between the biosensor's response in tissue and during calibration. However, when the biosensor response is linear our modeling suggests that, in principle, the calibration can be corrected by scaling factors: *Eq. 26* for agar, *Eq. 29* for tissue, and *Eq. 30* for tissue when the effects of the free diffusion space around the biosensor are significant. If the parameters of the scaling factors can be estimated or constrained, then this provides an approach for improved estimates of analyte concentrations, or bounds on concentrations, in tissue. It must be noted that the models do not imply that the concentration recorded in tissue is incorrect. The biosensors are indeed measuring the local concentration of the analyte; however, they have themselves reduced the local concentration through their measurement mechanism and this concentration differs from that in tissue far from the biosensor.

Biosensors could be designed to have less of an impact on the analyte in tissue, so that calibration and tissue conditions are better matched. This would require a lower total reaction rate for the enzyme in the biosensor, which would in turn result in a lower signal-to-noise ratio and a biosensor response potentially more dependent on the enzyme kinetics than the analyte concentration (Baronas et al. 2009).

Agar was used to provide a diffusive environment and denser concentration of agar could be considered as a means of altering the porosity or permeability. However, the diffusive parameters of agar are not greatly influenced by the density (McCabe 1972) and at higher densities the agar damaged the enzyme layer of the biosensor. Silica microbeads could also be mixed with the agar to create a controlled experimental model of excluded volume if desired: this would produce a stronger effect than that see in Fig. 4.

### Bath Application

When experiments are carried out with biosensors placed within tissue, the current produced by bath applying analyte is much smaller than that produced in free-flow conditions. For example, when adenosine is bath applied to the rat neocortex, there is reduction of 90% in the current measured in tissue compared with that observed in free-flow conditions (Wall and Richardson 2015) and when ATP is bath applied to the rat hippocampal slices only ∼5% could be detected, compared with free-flow calibration (Frenguelli et al. 2007). Such a difference between free-flow and tissue measurements supports the calibration mismatch proposed by our modeling. The effects of the density gradient produced in tissue by diffusion and tortuosity will be compounded by the presence of active removal mechanisms such as uptake into neurons and glia and metabolism. However, during adenosine application blocking nucleoside transport had only minor effects on the current measured in tissue (unpublished observations) suggesting that the effect maybe principally due to the density gradient set up by the biosensor.

### Existing Biosensor Models

Models of biosensors are typically applied to the case where the biosensor is placed in a well-stirred medium (Schulmeister 1990; Rinken and Tenno 2001; Baronas et al. 2009). Even in a well-stirred medium, a narrow layer around the biosensor will persist where transport is primarily due to diffusion and not convection. The substrate concentration will asymptotically increase towards the bulk concentration with distance from the biosensor. The Nernst diffusion layer approximation assumes transport in a region around the biosensor is solely due to diffusion; the thickness of this layer depends on the flow and viscosity of the medium. Consequently, it would not substantially effect the calibration signal as the flow in the bath is relatively rapid. Other modeling has examined the effects of substances that oxidize at the holding potential of the biosensor (+500 mV) and could be potential sources of positive or negative (antioxidants like ascorbic acid) interference (Lowry and O'Neill 1994; Lowry et al. 1994, 1998). To reduce the interference biosensors are coated in a permeable membrane and this can be modeled by an additional diffusion layer (Baronas et al. 2014). Modeling this additional layer around the biosensor is beyond the scope of the current paper, but its inclusion would not alter the paper's broad conclusions.

### Biosensor Geometry

Here we have considered a cylindrical design of biosensor often used in in experiments (Chen et al. 2002; Mikeladze et al. 2002; Shigetomi et al. 2013; Kotanen et al. 2014). The size of the biosensor has little impact on the calibration mismatch provided the thickness of the enzyme layer is greater than the typical distance the analyte diffuses before being oxidized (ℓ_b_), i.e., the biosensor is diffusion controlled. This work could be extended to consider other biosensor geometries such as discs (Chen et al. 1998; Kobayashi and Hoshi 2001; Razola et al. 2003; Patel et al. 2011), microelectrode arrays (Burmeister and Palmer 2003; Walker et al. 2007; Hascup et al. 2013), or twisted pairs of electrodes (Santos et al. 2015). While considering geometries is useful in optimizing design, such as for a plate-gap biosensor (Ivanauskas and Baronas 2008), and could help better quantify the discrepancy between free-flow and diffusive environments, it would not refute our key finding. Irrespective of the geometry, the consumption of the analyte by the biosensor will set up a concentration gradient resulting in lower signals in diffusive (tissue or agar) than in a free-flow calibration environment at equivalent concentrations.

### Multienzyme Biosensors

The idealized model of a biosensor considered here with a single-enzyme layer is likely to display the same characteristics as the more complex biosensors such as those that use a cascade of enzymes for adenosine or inosine (Llaudet et al. 2003; Wall and Richardson 2015; Frenguelli and Wall 2016), glutamate (Mikeladze et al. 2002), or acetylcholine (Chen et al. 1998). The main barrier for such analysis is the paucity of published information regarding the properties of the biosensors, which in many cases is proprietary. Additionally, there is substantial variation between biosensors in key properties such as enzyme layer thickness and reaction rate, which also change with repeated use. Accurate estimation of tissue concentrations would also necessitate more sophisticated models of the dynamics of the analyte and its breakdown quantities in tissue.

## GRANTS

The research was funded by Biotechnology and Biological Sciences Research Council (BBSRC) Grant BB/J0153691/1.

## DISCLOSURES

No conflicts of interest, financial or otherwise, are declared by the author(s).

## AUTHOR CONTRIBUTIONS

A.J.H.N., M.J.W., and M.J.E.R. analyzed data; A.J.H.N., M.J.W., and M.J.E.R. interpreted results of experiments; A.J.H.N. and M.J.E.R. prepared figures; A.J.H.N., M.J.W., and M.J.E.R. drafted manuscript; A.J.H.N., M.J.W., and M.J.E.R. edited and revised manuscript; A.J.H.N., M.J.W., and M.J.E.R. approved final version of manuscript; M.J.W. performed experiments.

## APPENDIX

The experimental conditions considered typically require the solution of two related second-order differential equations in cylindrical coordinates. The first is ∇^2^*A* = 0 using the radial operator (*Eq. 3*) and has general solution

(31) $$A\left(r\right)=c_{1}+c_{2}\;\log\left(r\right)$$

where *c*_1_ and *c*_2_ are constants determined by the boundary conditions. The second is *D*∇^2^*A* = *vA* and has a general solution in terms of zero-order modified Bessel functions

(32) $$A\left(r\right)=c_{3}I_{0}\;(\frac{r}{\ell })+c_{4}K_{0}\;(\frac{r}{\ell })$$

where *c*_3_ and *c*_4_ are constants determined by boundary conditions and ℓ, defined through ℓ^2^ = *D*/*v*, provides a scale for the distance the quantity of interest diffuses through the medium before being broken down. Given that part of the boundary conditions will require the matching of concentration gradients, the following relations for derivatives of Bessel functions are of use

(33) $$\frac{d}{dx}I_{0}\left(x\right)=I_{1}\left(x\right)\;\;\text{and}\;\;\frac{d}{dx}K_{0}\left(x\right)=-K_{1}\left(x\right)$$

where *I*_1_(*x*) and *K*_1_(*x*) are first-order modified Bessel functions. For large values of the argument *x*, the modified Bessel functions of order *n* can be approximated as follows

(34) $$I_{n}\left(x\right)≃\frac{1}{\sqrt{2\pi x}}e^{x}\;\;\text{and}\;\;K_{n}\left(x\right)≃\sqrt{\frac{\pi }{2x}}e^{-x}\;.$$

### Biosensor Calibration

The concentrations of the analyte *A*_b_ and breakdown product *H*_b_ in the biosensor enzyme layer obey *Eqs. 6* and *7*. In the steady state these become

(35) $$v_{\text{b}}\;A_{\text{b}}=D_{\text{Ab}}\nabla^{2}A_{\text{b}}$$

(36) $$0=D_{\text{Hb}}\nabla^{2}H_{\text{b}}+v_{\text{b}}\;A_{\text{b}}$$

with boundary conditions given by *Eqs. 8* and *9*.

#### Analyte concentration.

The solution of *Eq. 35* involves a length parameter ℓ_b_, defined through ℓ_b_^2^ = *D*_Ab_/*v*_b_, which provides a measure of the distance a molecule of analyte will diffuse into the biosensor enzyme layer before being broken down. The solution can be written

(37) $$A_{\text{b}}\left(r\right)=A_{2}X\left(r\right)$$

where for calibration the concentration *A*_2_ = *A*_b_(*r*_2_) at the biosensor surface at *r*_2_ is *A*_c_. The function *X*(*r*) is derived from *Eq. 32* and takes the form

(38) $$X\left(r\right)=\frac{K_{1}\;(\frac{r_{1}}{\ell_{\text{b}}})I_{0}\left(\frac{r}{\ell_{\text{b}}}\right)+I_{1}\left(\frac{r_{1}}{\ell_{\text{b}}}\right)K_{0}\left(\frac{r}{\ell_{\text{b}}}\right)}{K_{1}\left(\frac{r_{1}}{\ell_{\text{b}}}\right)I_{0}\left(\frac{r_{2}}{\ell_{\text{b}}}\right)+I_{1}\left(\frac{r_{1}}{\ell_{\text{b}}}\right)K_{0}\left(\frac{r_{2}}{\ell_{\text{b}}}\right)},$$

such that the analyte flux at the core, and therefore gradient of *A*_b_, are zero at *r*_1_ and, additionally, have been normalized so that *X*(*r*_2_) = 1. Provided the length that the analyte diffuses into the enzyme layer is much smaller than the size of biosensor core ℓ_b_ ≪ *r*_1_, the arguments of the modified Bessel functions will be large, so an asymptotic approximation (*Eq. 34*) can be used

(39) $$X\left(r\right)≃\sqrt{\frac{r_{2}}{r}}\frac{\cosh\left(\frac{r-r_{1}}{\ell_{\text{b}}}\right)}{\cosh\left(\frac{r_{2}-r_{1}}{\ell_{\text{b}}}\right)}.$$

#### Breakdown product concentration and flux.

The solution for *H*_b_ can be separated into two components

(40) $$H_{b}=h_{b}-\frac{D_{\text{A}}}{D_{\text{H}}}A_{\text{b}\;}\;\text{where}\;\;\nabla^{2}h_{\text{b}}=0.$$

Applying the boundary condition *H*_b_(*r*_1_) = 0 at the core and using the solution given in *Eq. 31* allows *h*_b_ to be written in the form

(41) $$h_{b}\left(r\right)=\frac{D_{\text{A}}}{D_{\text{H}}}\left(\gamma \frac{\log\left(r/r_{1}\right)}{\log\left(r_{2}/r_{1}\right)}\left(A_{2}-A_{1}\right)+A_{1}\right)$$

where *A*_1_ = *A*_b_(*r*_1_) can be found from *Eq. 37* and is a constant. In the calibration case, using the condition at the biosensor surface *H*_b_(*r*_2_) = 0, gives = 1 so that

(42) $$H_{b}\left(r\right)=\frac{D_{\text{A}}}{D_{\text{H}}}\left(\frac{\log\left(r/r_{1}\right)}{\log\left(r_{2}/r_{1}\right)}\left(A_{2}-A_{1}\right)-\left(A_{\text{b}}\left(r\right)-A_{1}\right)\right).$$

The flux (and therefore gradient) of the analyte at *r*_1_ is zero so the flux of the breakdown product at *r*_1_ is simply

(43) $$J_{\text{H}}=-\frac{\alpha_{\text{b}}\theta_{\text{b}}D_{\text{A}}}{r_{1}\log\left(r_{2}/r_{1}\right)}\left(A_{2}-A_{1}\right)$$

so the current for a length *z*_b_ of biosensor takes the form

(44) $$I=2F\frac{2\pi z_{b}\alpha_{\text{b}}\theta_{\text{b}}D_{\text{A}}}{\log\left(r_{2}/r_{1}\right)}\left(A_{2}-A_{1}\right).$$

Note this does not depend on the diffusion coefficient of H_2_O_2_ in the biosensor enzyme layer. When *r*_2_ − *r*_1_ ≫ ℓ_b_, meaning the analyte is almost certainly broken down before reaching the core, the biosensor is said to be diffusion limited (Baronas et al. 2009) as the current no longer depends on *v*_b_. In this case *A*_2_ ≫ *A*_1_ and the current may be approximated as

(45) $$I≃2F\frac{2\pi z_{b}\alpha_{\text{b}}\theta_{\text{b}}D_{\text{A}}}{\log\left(r_{2}/r_{1}\right)}A_{\text{c}}^{*}$$

where the boundary condition *A*_b_(*r*_2_) = $A_{c}^{*}$ has been used.

### Biosensor in Agar

In cylindrical geometry the agar region extends from *r*_2_ → *r*_3_ within which the steady-state equations

$$D_{\text{Ag}}\nabla^{2}A_{\text{g}}=0\;\text{and}\;D_{\text{Hg}}\nabla^{2}H_{\text{g}}=0$$

are obeyed by the concentrations. At the agar surface *r*_3_ the analyte has a fixed concentration $A_{g}^{*}$ and the breakdown product has zero concentration, corresponding to free-flow bath conditions. Within the biosensor the concentrations obey formulas in *Eqs. 35* and *36* with continuity of concentration and flux across the biosensor-agar interface at *r*_2_. Note that these conditions allow for loss and diffusion of H_2_O_2_ from the biosensor into the agar and hence require a different boundary condition at *r*_2_ to the zero *H*_b_(*r*_2_) used for the calibration case.

#### Analyte concentration.

Within the biosensor enzyme layer *A*_b_ has the same functional form as *Eq. 37* except that here the concentration *A*_2_ at *r*_2_ is a fraction *c*_g_ of $A_{g}^{*}$ so that *A*_2_ = $c_{g}A_{g}^{*}$ Within agar the solution takes the form as *Eq. 31*, which, on matching concentrations at the biosensor surface *r*_2_, can be written

(46) $$A_{g}\left(r\right)=A_{\text{g}}^{*}\left(1-\left(1-c_{g}\right)\frac{\log\left(r_{3}/r\right)}{\log\left(r_{3}/r_{2}\right)}\right).$$

Matching the analyte fluxes provides an equation for *c*_g_

(47) $$\alpha_{\text{b}}D_{\text{Ab}}c_{\text{g}}\frac{dX}{dr}|{}_{r_{2}}=\alpha_{g}D_{\text{Ag}}\frac{\left(1-c_{\text{g}}\right)}{r_{2}\,\;\log\left(r_{3}/r_{2}\right)}.$$

This can be rearranged to find the constant

(48) $$c_{\text{g}}=\frac{1}{1+\frac{\alpha_{\text{b}}\theta_{\text{b}}}{\alpha_{g}\theta_{g}}{X\prime }^{}\left(r_{2}\right)r_{2}\log\left(r_{3}/r_{2}\right)}$$

which solves the problem for the analyte *A*. Provided the length scale ℓ_b_ is smaller than the biosensor core radius (ℓ_b_ ≪ *r*_1_), the same expansion used (*Eq. 39*) shows *X′*(*r*_2_) ≃$\frac{1}{\ell_{\text{b}}}$, which gives the result (*Eq. 23*).

#### Breakdown product concentration and flux.

The concentration *H*_b_ within the biosensor takes the form given by *Eq. 41*. However, the concentration at the biosensor surface is nonzero because *H* is not washed away but diffuses into the agar. The analyte concentration appears in *Eq. 40* for the biosensor and so, to simplify the boundary matching at *r*_2_, it proves convenient to add the analyte concentration within agar also

(49) $$H_{\text{g}}=h_{\text{g}}-\frac{D_{\text{A}}}{D_{\text{H}}}A_{\text{g}\;}\;\text{where}\;\;\nabla^{2}h_{\text{g}}=0.$$

Boundary conditions related to the analyte component are automatically matched so just the components *h*_b_ and *h*_g_ remain to be matched. At *r*_1_ and *r*_3_ the following boundary conditions hold

(50) $$h_{\text{b}}\left(r_{1}\right)=\frac{D_{\text{A}}}{D_{\text{H}}}A_{1\;}\;\text{and}\;\;h_{\text{g}}\left(r_{3}\right)=\frac{D_{\text{A}}}{D_{\text{H}}}A_{\text{g}}^{*}$$

constraining the *Eq. 31* solutions to give

(51) $$h_{\text{b}}\left(r\right)=\frac{D_{\text{A}}}{D_{\text{H}}}\left(A_{1}+\kappa_{b}\left(A_{\text{g}}^{*}-A_{1}\right)\frac{\log\left(r/r_{1}\right)}{\log\left(r_{2}/r_{1}\right)}\right)$$

(52) $$h_{\text{g}}\left(r\right)=\frac{D_{\text{A}}}{D_{\text{H}}}\left(A_{\text{g}}^{*}-\kappa_{g}\left(A_{\text{g}}^{*}-A_{1}\right)\frac{\log\left(r_{3}/r\right)}{\log\left(r_{3}/r_{2}\right)}\right).$$

The constants κ_b_ and κ_g_ are fixed by matching conditions at the biosensor-agar interface at *r*_2_. The concentration match requires *h*_b_(*r*_2_) = *h*_g_(*r*_2_) and the flux match $\alpha_{b}D_{Hb}h_{b}^{\prime }\left(r_{2}\right)=$$\alpha_{g}D_{Hg}h_{g}^{\prime }\left(r_{2}\right)$ resulting in

(53) $$1=\kappa_{\text{b}}+\kappa_{\text{g}}\;\text{and}\;\;\frac{\alpha_{\text{b}}\theta_{\text{b}}\kappa_{\text{b}}}{\log\left(r_{2}/r_{1}\right)}=\frac{\alpha_{\text{g}}\theta_{\text{g}}\kappa_{\text{g}}}{\log\left(r_{3}/r_{2}\right)}.$$

Solving for the constants gives

(54) $$\kappa_{\text{b}}=\frac{1}{1+\frac{\alpha_{\text{b}}\theta_{\text{b}}}{\alpha_{\text{g}}\theta_{\text{g}}}\frac{\log\left(r_{3}/r_{2}\right)}{\log\left(r_{2}/r_{1}\right)}}\;\text{and}\;\;\kappa_{\text{g}}=\frac{1}{1+\frac{\alpha_{\text{g}}\theta_{\text{g}}}{\alpha_{\text{b}}\theta_{\text{b}}}\frac{\log\left(r_{2}/r_{1}\right)}{\log\left(r_{3}/r_{2}\right)}}.$$

The *H* flux at the biosensor core is

(55) $$J_{\text{H}}=-\alpha_{\text{b}}D_{\text{Hb}}\frac{\partial h_{\text{b}}}{\partial r}|{}_{r_{1}}=-\frac{\alpha_{\text{b}}\theta_{\text{b}}D_{\text{A}}}{r_{1}\log\left(r_{2}/r_{1}\right)}\left(A_{\text{g}}^{*}-A_{1}\right)\kappa_{\text{b}}.$$

This gives the following form for the current

(56) $$I=2F\frac{2\pi z_{\text{b}}\alpha_{\text{b}}\theta_{\text{b}}D_{\text{A}}}{\log\left(r_{2}/r_{1}\right)}\left(A_{\text{g}}^{*}-A_{1}\right)\kappa_{\text{b}}.$$

We can now compare this form with the current for the calibration condition (*Eq. 44*). In terms of the function *X*(*r*) we have for the agar case ${\mathrm{(A}}_{g}^{*}$ − *A*_1_) = $A_{\text{g}}^{*}$ (1 − *c*_g_*X*_1_) where

(57) $$c_{\text{g}}=\frac{1}{1+\frac{\alpha_{\text{b}}\theta_{\text{b}}}{\alpha_{\text{g}}\theta_{\text{g}}}X^{\prime }\left(r_{2}\right)r_{2}\;\log\left(r_{3}/r_{2}\right)}$$

for the calibration case we have (*A*_2_ − *A*_1_) = $A_{\text{c}}^{*}$ (1 − *X*_1_) Dividing out the current for the agar condition *I*_agar_ and calibration *I*_cal_ gives the ratio

(58) $$\frac{I_{\text{agar}}}{I_{\text{cal}}}=\frac{A_{\text{g}}^{*}}{A_{c}^{*}}\frac{\left(1-c_{\text{g}}X_{1}\right)}{\left(1-X_{1}\right)}\kappa_{b}$$

which can be rearranged to provide a formula for the calibration mismatch

(59) $$A_{\text{inferred}}=A_{c}^{*}\frac{I_{\text{agar}}}{I_{\text{cal}}}=A_{\text{g}}^{*}\frac{\left(1-c_{\text{g}}X_{1}\right)}{\left(1-X_{1}\right)}\kappa_{b}$$

and hence the concentration measured is a factor

(60) $$\frac{\left(1-c_{\text{g}}X_{1}\right)}{\left(1-X_{1}\right)}\kappa_{b}$$

of the concentration $A_{g}^{*}$ at the outer surface of the agar block. Note that for diffusion-limited biosensors, where *A*_2_ ≫ *A*_1_ we have *X*_1_ ≪ 1 and so *A*_inferred_ ≃ $A_{g}^{*}\kappa_{b}$ is a good approximation.

### Biosensor in Tissue

In tissue the analyte is modeled as having a steady-state extracellular concentration *A* due to the balance between production and removal rates. In the steady-state the analyte dynamics (*Eq. 15*) reduce to

(61) $$D_{\text{At}}\nabla^{2}A_{\text{t}}=v_{\text{t}}\left(A_{\text{t}}-A_{t}^{*}\right).$$

The breakdown product H_2_O_2_ is considered to be removed very rapidly from tissue and so has zero concentration outside the biosensor. The boundary conditions at the biosensor-tissue interface at *r*_2_ are given by *Eq. 16* with the conditions at the biosensor core given by *Eq. 8*.

#### Analyte concentration.

Within the biosensor the functional form is given by *Eq. 37* with the concentration at the biosensor surface a fraction *c*_t_ of $A_{t}^{*}$ so *A*_2_ = *c*_t_ $A_{\mathrm{t.}}^{*}$ Within the tissue the limit *r* → ∞ should remain finite so the solution is constructed from the zero-order modified Bessel function *K*_0_(*r/*ℓ_t_)

(62) $$A_{\text{t}}=A_{t}^{*}\left(1-\left(1-c_{\text{t}}\right)Y\left(r\right)\right)\;\text{with}\;Y\left(r\right)=\frac{K_{0}\left(\frac{r}{\ell_{t}}\right)}{K_{0}\left(\frac{r_{2}}{\ell_{t}}\right)}$$

where ℓ_t_ is a length scale defined through *ℓ*_t_^2^ = *D*_At_/*v*_t_. The function *Y* (*r*) has been normalized such that *Y* (*r*_2_) = 1 so concentrations are already matched at *r*_2_ and it remains only to render the flux continuous

(63) $$\alpha_{\text{b}}D_{\text{Ab}}c_{\text{t}}\frac{dX}{dr}|{}_{r_{2}}=-\alpha_{\text{t}}D_{\text{At}}\left(1-c_{t}\right)\frac{dY}{dr}|{}_{r_{2}}.$$

This can be rearranged to give

(64) $$c_{\text{t}}={\left(1-\frac{\alpha_{\text{b}}\theta_{\text{b}}}{\alpha_{\text{t}}\theta_{\text{t}}}\,\frac{{X\prime }^{}\left(r_{2}\right)}{{Y\prime }^{}\left(r_{2}\right)}\,\right)}^{-1}$$

for the fractional reduction of the bulk concentration $A_{t}^{*}$ the surface of the biosensor due to the combined effects of diffusive transport and analyte removal by the biosensor. Note this fraction is strictly positive because the gradient of *Y*(*r*) is negative. The gradients required take the form

(65) $$\ell_{\text{b}}{X\prime }^{}\left(r_{2}\right)=\frac{K_{1}\;(\frac{r_{1}}{\ell_{\text{b}}})I_{1}\left(\frac{r_{2}}{\ell_{\text{b}}}\right)-I_{1}\left(\frac{r_{1}}{\ell_{\text{b}}}\right)K_{1}\left(\frac{r_{2}}{\ell_{\text{b}}}\right)}{K_{1}\;(\frac{r_{1}}{\ell_{\text{b}}})I_{0}\left(\frac{r_{2}}{\ell_{\text{b}}}\right)+I_{1}\left(\frac{r_{1}}{\ell_{\text{b}}}\right)K_{0}\left(\frac{r_{2}}{\ell_{\text{b}}}\right)}$$

for *X* and for the function *Y* we have

(66) $$\ell_{\text{t}}{\mathrm{Y\prime}}^{}\left(r_{2}\right)=-\frac{K_{1}\;(\frac{r_{2}}{\ell_{\text{t}}})}{K_{0}\;(\frac{r_{2}}{\ell_{\text{t}}})}.$$

#### Breakdown product concentration and flux.

Because the H_2_O_2_ concentration in tissue is zero, the boundary conditions on *H* are the same as for the calibration case, except that instead of $A_{c}^{*}$ we have ${c_{t}A}_{t}^{*}$ for the concentration at the biosensor surface. The concentration of the breakdown product and the biosensor current take the same forms as *Eqs. 42* and *44*, respectively, for the calibration case but now calculated by replacing $A_{c}^{*}\;with$ $c_{\text{t}}A_{t}^{*}$ throughout. Hence, the ratio of the currents in calibration and tissue conditions used to infer the concentration would give an erroneous result

(67) $$A_{\text{inferred}}=A_{\text{c}}^{*}\frac{I_{\text{tis}}}{I_{\text{cal}}}=c_{\text{t}}{\;A}_{\text{t}}^{*}$$

underestimating the true extracellular concentration $A_{t}^{*}$ by a factor *c*_t_ given by *Eq. 64*.

### Biosensor in Tissue with Free Space

To model the effect of damage due to the insertion of the biosensor, a scenario in which there is an additional free diffusion space between the biosensor surface at *r*_2_ out to a radius *r*_s_ is considered. The tissue continues as before at radii greater than *r*_s_. Within the free space there is unimpeded diffusion so that in the steady state

(68) $$D_{\text{A}}\nabla^{2}A_{\text{s}}=0\;\text{and}\;D_{\text{H}}\nabla^{2}H_{\text{s}}=0$$

where here the free diffusion coefficients are used and there is no tortuosity or porosity.

#### Analyte concentration.

We can adapt the solutions for *A* used previously so that

(69) $$A_{\text{b}}=A_{\text{t}}^{*}\kappa_{b}\;X\left(r\right),$$

(70) $$A_{\text{s}}=A_{\text{t}}^{*}\left(\kappa_{b}+\kappa_{s}\frac{\log\left(r/r_{2}\right)}{\log\left(r_{s}/r_{2}\right)}\right)$$

(71) $$A_{t}=A_{\text{t}}^{*}\left(1-\kappa_{t}Z\left(r\right)\right)$$

where *X*(*r*) is as before and *Z*(*r*) = *K*_0_$(\frac{r}{\ell_{\text{t}}})/$*K*_0_$\left(\frac{r_{s}}{\ell_{\text{t}}}\right)$ so that *Z*(*r*_s_) = 1. The concentrations are already matched at *r*_2_ and at *r*_s_ we have the condition 1 = κ_b_ + κ_s_ + κ_t_. The flux conditions at *r*_2_ and at *r*_s_ give

(72) $$\kappa_{b}=\frac{\kappa_{s}}{\alpha_{\text{b}}\theta_{\text{b}}X^{\prime }\left(r_{2}\right)r_{2}\;\log\left(r_{s}/r_{2}\right)},$$

(73) $$\kappa_{t}=\frac{-\kappa_{s}}{\alpha_{\text{t}}\theta_{\text{t}}Z^{\prime }\left(r_{s}\right)r_{s}\;\log\left(r_{s}/r_{2}\right)}.$$

So that

(74) $$\begin{array}{l} \frac{1}{\kappa_{s}}=1+\frac{1}{\alpha_{\text{b}}\theta_{\text{b}}X^{\prime }\left(r_{2}\right)r_{2}\;\log\left(\frac{r_{s}}{r_{2}}\right)}\\ \;\;-\frac{1}{\alpha_{t}\theta_{\text{t}}Z^{\prime }\left(r_{s}\right)r_{s}\log\left(\frac{r_{s}}{r_{2}}\right)} \end{array}$$

from which the other constants follow, and in particular

(75) $$\frac{1}{\kappa_{\text{b}}}=1+\alpha_{\text{b}}\theta_{\text{b}}r_{2}X\prime \left(r_{2}\right)\log\left(\frac{r_{s}}{r_{2}}\right)-\frac{\alpha_{\text{b}}\theta_{\text{b}}}{\alpha_{\text{t}}\theta_{\text{t}}}\frac{r_{2}X\prime \left(r_{2}\right)}{r_{s}Z\prime \left(r_{s}\right)}$$

will be required later for the mismatch factor.

#### Breakdown product concentration.

The concentration for the quantity *H* is straightforward to derive because *H*_t_ = 0. In the biosensor enzyme layer and free-diffusion region

(76) $$H_{\text{b}}=\frac{D_{\text{A}}}{D_{\text{H}}}\left(\gamma_{\text{b}}\left(A_{2}-A_{1}\right)\frac{\log\left(r/r_{1}\right)}{\log\left(r_{2}/r_{1}\right)}-\left(A_{\text{b}}\left(r\right)-A_{1}\right)\right)$$

and

(77) $$H_{\text{s}}=\gamma_{\text{s}}\left(A_{2}-A_{1}\right)\frac{D_{\text{A}}}{D_{\text{H}}}\left(1-\frac{\log\left(r/r_{2}\right)}{\log\left(r_{s}/r_{2}\right)}\right).$$

From matching these at *r*_2_ we get γ_b_ − 1 = γ_s_ and

(78) $$\alpha_{\text{b}}\theta_{\text{b}}\left(\frac{\gamma_{\text{b}}}{r_{2}\;\log\left(r_{2}/r_{1}\right)}-\frac{A_{\text{b}}^{\prime }\left(r_{2}\right)}{A_{2}-A_{1}}\right)=\frac{-\gamma_{\text{s}}}{r_{2}\;\log\left(r_{s}/r_{2}\right)}.$$

So that

(79) $$\gamma_{\text{b}}=\frac{1+\alpha_{\text{b}}\theta_{\text{b}}r_{2}\log\left(r_{s}/r_{2}\right)\frac{{A^{\prime }}_{b}\left(r_{2}\right)}{A_{2}-A_{1}}}{1+\alpha_{\text{b}}\theta_{\text{b}}\frac{\log\left(r_{s}/r_{2}\right)}{\log\left(r_{2}/r_{1}\right)}}.$$

Finally, the flux for *H* onto the biosensor becomes

(80) $$J_{\text{H}}≃-\frac{\alpha_{\text{b}}\theta_{\text{b}}D_{\text{A}}}{r_{1}\;\log\left(r_{2}/r_{1}\right)}\left(A_{2}-A_{1}\right)\gamma_{\text{b}}$$

from which the current follows. For the calibration case we have (*A*_2_ − *A*_1_) = $A_{\text{c}}^{*}$ (*X*_2_ − *X*_1_) whereas for the tissue case we have *A*_2_ − *A*_1_ = $A_{t}^{*}\;\mathrm{\kappa b}$ (*X*_2_ − *X*_1_) with the quantities *X*_1_ and *X*_2_ unchanged for the two cases. Hence, the ratio of the currents yields a combined factor κ_b_γ_b_ such that

(81) $$A_{\text{inferred}}=A_{\text{c}}^{*}\frac{I_{\text{tis}}}{I_{\text{cal}}}=\kappa_{\text{b}}\gamma_{\text{b}}A_{\text{t}}^{*}$$

where κ_b_ and γ_b_ are given by *Eqs. 75* and *79*.

## References

[B1] Baronas R, Kulys J, Lancinskas A, Zilinskas A Effect of diffusion limitations on multianalyte determination from biased biosensor response. Sensors 14: 4634–4656, 2014. 24608006 10.3390/s140304634 PMC4003961

[B2] Baronas R, Ivanauskas F, Kulys J Mathematical Modeling of Biosensors: an Introduction for Chemists and Mathematicians. New York: Springer, 2009.

[B3] Bélanger M, Allaman I, Magistretti PJ Brain energy metabolism: focus on astrocyte-neuron metabolic cooperation. Cell Metab 14: 724–738, 2011. 22152301 10.1016/j.cmet.2011.08.016

[B4] Berti V, Vanzi E, Polito C, Pupi A Back to the future: the absolute quantification of cerebral metabolic rate of glucose. Clin Trans Imaging 1: 289–296, 2013.

[B5] Betz AL Identification of hypoxanthine transport and xanthine oxidase activity in brain capillaries. J Neurochem 44: 574–579. 3838099 10.1111/j.1471-4159.1985.tb05451.x

[B6] Bruno JP, Gash C, Martin B, Zmarowski A, Pomerleau F, Burmeister J, Huettl P, Gerhardt GA Second-by-second measurement of acetylcholine release in prefrontal cortex. Eur J Neurosci 24: 2749–2757, 2006. 17156201 10.1111/j.1460-9568.2006.05176.x

[B7] Burmeister JJ, Palmer MA Ceramic-based multisite microelectrode array for rapid choline measures in brain tissue. Anal Chim Acta 481: 65–74, 2003.

[B8] Cambiaso A, Delfino L, Grattarola M, Verreschi G, Ashworth D, Maines A, Vadgama P Modelling and simulation of a diffusion limited glucose biosensor. Sensors Actuators B: Chem 33: 203–207, 1996.

[B9] Chen Q, Kobayashi Y, Takeshita H, Hoshi T, Anzai J Avidin-biotin system-based enzyme multilayer membranes for biosensor applications: optimization of loading of choline esterase and choline oxidase in the bienzyme membrane for acetylcholine biosensors. Electroanalysis 10: 94–97, 1998.

[B10] Chen X, Matsumoto N, Hu Y, Wilson GS Electrochemically mediated electrodeposition/electropolymerization to yield a glucose microbiosensor with improved characteristics. Anal Chem 74: 368–372, 2002. 11811410 10.1021/ac015628m

[B11] Clarke DD, Sokoloff L Regulation of cerebral metabolic rate. In: Basic Neurochemistry (6th ed). Philadeliphia, PA: Lippincott-Raven, 1999.

[B12] Coche-Guerente L, Cosnier S, Innocent C, Mailley P Development of amperometric biosensors based on the immobilization of enzymes in polymer films electrogenerated from a series of amphiphilic pyrrole derivatives. Anal Chim Acta 311: 23–30, 1995.

[B13] Dale N Measurement of purine release with microelectrode biosensors. In: Microelectrode Biosensors. New York: Humana, 2013, p. 221–240.

[B14] Dale N, Hatz S, Tian F, Llaudet E Listening to the brain: microelectrode biosensors for neurochemicals. Trends Biotechnol 23: 420–428, 2005. 15950302 10.1016/j.tibtech.2005.05.010

[B15] Dash MB, Bellesi M, Tononi G, Cirelli C Sleep/wake dependent changes in cortical glucose concentrations. J Neurochem 124: 79–89, 2013. 23106535 10.1111/jnc.12063 PMC3518620

[B16] Frayling C, Britton R, Dale N ATP-mediated glucosensing by hypothalamic tanycytes. J Physiol 589: 2275–2286, 2011. 21486800 10.1113/jphysiol.2010.202051 PMC3098703

[B17] Frenguelli BG, Wigmore G, Llaudet E, Dale N Temporal and mechanistic dissociation of ATP and adenosine release during ischaemia in the mammalian hippocampus. J Neurochem 101: 1400–1413, 2007. 17459147 10.1111/j.1471-4159.2006.04425.x PMC1920548

[B18] Frenguelli BG, Wall MJ Combined electrophysiological and biosensor approaches to study purinergic regulation of epileptiform activity in cortical tissue. J Neurosci Methods 260: 202–214, 2016. 26381061 10.1016/j.jneumeth.2015.09.011

[B19] Garcia-Belmonte G Effect of electrode morphology on the diffusion length of the doping process of electronically conducting polypyrrole films. Electrochem Comm 5: 236–240, 2003.

[B20] Gourine AV, Dale N, Korsak A, Llaudet E, Tian F, Huckstepp R, Spyer KM Release of ATP and glutamate in the nucleus tractus solitarii mediate pulmonary stretch receptor (Breuer-Hering) reflex pathway. J Physiol 586: 3963–3978, 2008. 18617567 10.1113/jphysiol.2008.154567 PMC2538935

[B21] Hallik A, Alumaa A, Kurig H, Janes A, Lust E, Tamm J On the porosity of polypyrrole films. Synthetic Metals 157: 1085–1090, 2007.

[B22] Hascup KN, Hascup ER, Littrell OM, Hinzman JM, Werner CE, Davis VA, Burmeister JJ, Pomerleau F, Quintero JE, Huettl P, Herhardt GA Microelectrode array fabrication and optimization for selective neurochemical detection. In: Microelectrode Biosensors. New York: Humana, 2013, p. 27–54, 2013.

[B23] Hashimoto S A new spectrophotometric assay method of xanthine oxidase in crude tissue homogenate. Anal Biochem 62: 426–435, 1974. 4441740 10.1016/0003-2697(74)90175-4

[B24] Hrabe J, Hrabetova S, Segeth KA model of effective diffusion and tortuosity in the extracellular space of the brain. Biophys J 87: 1606–1617, 2004. 15345540 10.1529/biophysj.103.039495 PMC1304566

[B25] Hu Y, Mitchell KM, Albahadily FN, Michaelis EK, Wilson GS Direct measurement of glutamate release in the brain using a dual enzyme-based electrochemical sensor. Brain Res 659: 117–125, 1994. 7820652 10.1016/0006-8993(94)90870-2

[B26] Ivanauskas F, Baronas R Numerical simulation of a plate-gap biosensor with an outer porous membrane. Simulation Model Practice Theory 16: 962–970, 2008.

[B27] Ivanauskas F, Kaunietis I, Laurinavicius V, Razumiene J, Simkus R Apparent Michaelis constant of the enzyme modified porous electrode. J Math Chem 43: 1516–1526, 2008.

[B28] Klyuch BP, Dale N, Wall MJ Deletion of ecto5-nucleotidase (CD73) reveals direct action potential dependent adenosine release. J Neurosci 32: 3842–3847, 2012. 22423104 10.1523/JNEUROSCI.6052-11.2012 PMC6703466

[B29] Kobayashi Y, Hoshi T Glucose and lactate biosensors prepared by a layer-by-layer deposition of concanavalin A and mannose-labeled enzymes: electrochemical response in the presence of electron mediators. Chem Pharmaceutical Bull 49: 755–757, 2001. 10.1248/cpb.49.755 11411531

[B30] Kotanen CN, Karunwi O, Guiseppi-Elie A Biofabrication using pyrrole electropolymerization for the immobilization of glucose oxidase and lactate oxidase on implanted microfabricated biotransducers. Bioengineering 1: 85–110, 2014. 10.3390/bioengineering1010085 28955018

[B31] Lalo U, Palygin O, Rasooli-Nejad S, Andrew J, Haydon PG, Pankratov Y Exocytosis of ATP from astrocytes modulates phasic and tonic inhibition in the neocortex. PLoS Biol 12: e1001747, 2014. 24409095 10.1371/journal.pbio.1001747 PMC3883644

[B32] Llaudet E, Botting NP, Crayston JA, Dale N A three-enzyme microelectrode sensor for detecting purine release from central nervous system. Biosens Bioelectron 18: 43–52, 2003. 12445443 10.1016/s0956-5663(02)00106-9

[B33] Llaudet E, Hatz S, Droniou M, Dale N Microelectrode biosensor for real-time measurement of ATP in biological tissue. Anal Chem 77: 3267–3273, 2005. 15889918 10.1021/ac048106q

[B34] Lopatář J, Dale N, Frenguelli BG Pannexin1-mediated ATP release from area CA3 drives mGlu5 dependent neuronal oscillations. Neuropharmacology 93: 219–228, 2015. 25645390 10.1016/j.neuropharm.2015.01.014

[B35] Lowry JP, McAteer K, El Atrash SS, Duff A, O'Neill RD Characterization of glucose oxidasemodified poly (phenylenediamine)-coated electrodes in vitro and in vivo: homogeneous interference by ascorbic acid in hydrogen peroxide detection. Anal Chem 66: 1754–1761, 1994.

[B36] Lowry JP, O'Neill RD Partial characterization in vitro of glucose oxidase-modified poly (phenylenediamine)-coated electrodes for neurochemical analysis in vivo. Electroanalysis 6: 369–379, 1994.

[B37] Lowry JP, Miele M, O'Neill RD, Boutelle MG, Fillenz M An amperometric glucose-oxidase/poly (ophenylenediamine) biosensor for monitoring brain extracellular glucose: in vivo characterisation in the striatum of freely-moving rats. J Neurosci Methods 79: 65–74, 1998. 9531461 10.1016/s0165-0270(97)00171-4

[B38] McCabe M The diffusion coefficient of caffeine through agar gels containing a hyaluronic acid protein complex. A model system for the study of the permeability of connective tissues. Biochem J 127: 249–253, 1972. 5073746 10.1042/bj1270249 PMC1178579

[B39] Mikeladze E, Collins A, Sukhacheva M, Netrusov A, Csoregi E Characterization of a glutamate biosensor based on a novel glutamate oxidase integrated into a redox hydrogel. Electroanalysis 14: 1052–1059, 2002.

[B40] Monda MS, Mitra S Kinetics and thermodynamics of the molecular mechanism of the reductive half-reaction of xanthine oxidase. Biochemistry 33: 10305–10312, 1994. 8068667 10.1021/bi00200a010

[B41] Nicholson CH, Phillips JM, Gardner-Medwin AR Diffusion from an iontophoretic point source in the brain: role of tortuosity and volume fraction. Brain Res 169: 580–584, 1979. 445169 10.1016/0006-8993(79)90408-6

[B42] Oldenziel WH, Dijkstra G, Cremers TI, Westerink BH In vivo monitoring of extracellular glutamate in the brain with a microsensor. Brain Res 1118: 34- 42, 2006. 16956598 10.1016/j.brainres.2006.08.015

[B43] Patel BA, Rogers M, Wieder T, O'Hare D, Boutelle MG ATP microelectrode biosensor for stable long-term in vitro monitoring from gastrointestinal tissue. Biosens Bioelectron 26: 2890–2896, 2011. 21163639 10.1016/j.bios.2010.11.033

[B44] Razola SS, Pochet S, Grosfils K, Kauffmann J Amperometric determination of choline released from rat submandibular gland acinar cells using a choline oxidase biosensor. Biosens Bioelectron 18: 185–191, 2003. 12485764 10.1016/s0956-5663(02)00186-0

[B45] Reivich M, Alavi A, Wolf A, Fowler J, Russell J, Arnett C, MacGregor R, Shiue C, Atkins H, Anand A, Dann R, Greenberg JH Glucose metabolic rate kinetic model parameter determination in humans: the lumped constants and rate constants for [18F] uorodeoxyglucose and [11C] deoxyglucose. J Cereb Blood Flow Metab 5: 179–192, 1985. 3988820 10.1038/jcbfm.1985.24

[B46] Rinken T, Tenno T Dynamic model of amperometric biosensors. Characterisation of glucose biosensor output. Biosens Bioelectron 16: 53–59, 2001. 11261853 10.1016/s0956-5663(00)00133-0

[B47] Santos RM, Laranjinha J, Sirota A Simultaneous measurement of cholinergic tone and neuronal network dynamics in vivo in the rat brain using a novel choline oxidase based electrochemical biosensor. Biosens Bioelectron 69: 83–94, 2015. 25706061 10.1016/j.bios.2015.02.003

[B48] Schulmeister T Mathematical modelling of the dynamic behaviour of amperometric enzyme electrodes. Select Electrode Rev 12: 203–260, 1990.

[B49] Shigetomi E, Jackson-Weaver O, Huckstepp RT, O'Dell TJ, Khakh BS TRPA1 channels are regulators of astrocyte basal calcium levels and long-term potentiation via constitutive D-serine release. J Neurosci 33: 10143–10153, 2013. 23761909 10.1523/JNEUROSCI.5779-12.2013 PMC3682388

[B50] Simelevicius D, Baronas R Computational modelling of amperometric biosensors in the case of substrate and product inhibition. J Math Chem 47: 430–445, 2010.

[B51] Simelevicius D, Baronas R Mechanisms controlling the sensitivity of amperometric biosensors in the case of substrate and product inhibition. In: SIMUL 2011, The Third International Conference on Advances in System Simulation. Barcelona, Spain: Iaria, 2011, p. 61–66.

[B52] Simelevicius D, Baronas R, Kulys J Modelling of amperometric biosensor used for synergistic substrates determination. Sensors 12: 4897–4917, 2012. 22666066 10.3390/s120404897 PMC3355448

[B53] Stikoniene O, Ivanauskas F, Laurinavicius V The influence of external factors on the operational stability of the biosensor response. Talanta 81: 1245–1249, 2010. 20441891 10.1016/j.talanta.2010.02.016

[B54] van Stroe-Biezen SA, Everaerts FM, Janssen LJ, Tacken RA Diffusion coefficients of oxygen, hydrogen peroxide and glucose in a hydrogel. Anal Chim Acta 273: 553–560, 1993.

[B55] Sykova E, Nicholson C Diffusion in brain extracellular space. Physiol Rev 88: 1277–1340, 2008. 18923183 10.1152/physrev.00027.2007 PMC2785730

[B56] Tian F, Gourine AV, Huckstepp RT, Dale N A microelectrode biosensor for real time monitoring of l-glutamate release. Anal Chim Acta 645: 86–91, 2009. 19481635 10.1016/j.aca.2009.04.048

[B57] Updike SJ, Hicks GP The enzyme electrode. Nature 214: 986–988, 1967. 6055414 10.1038/214986a0

[B58] Van Gompel JJ, Bower MR, Worrell GA, Stead M, Chang SY, Goerss SJ, Kim I, Bennet KE, Meyer FB, Marsh WR, Blaha CD Increased cortical extracellular adenosine correlates with seizure termination. Epilepsia 55: 233–244, 2014. 24483230 10.1111/epi.12511 PMC4104491

[B59] Walker E, Wang J, Hamdi N, Monbouquette HG, Maidment NT Selective detection of extracellular glutamate in brain tissue using microelectrode arrays coated with over-oxidized poly pyrrole. Analyst 132: 1107–1111, 2007. 17955144 10.1039/b706880h

[B60] Wall MJ, Richardson MJ Localised adenosine signaling provides fine-tuned negative feedback over a wide dynamic range of neocortical network activities. J Neurophysiol 113: 871–882, 2015. 25392170 10.1152/jn.00620.2014 PMC4312871

[B61] Wells JA, Christie IN, Hosford PS, Huckstepp RT, Angelova PR, Vihko P, Cork SC, Abramov AY, Teschemacher AG, Kasparov S, Lythgoe MF A critical role for purinergic signaling in the mechanisms underlying generation of BOLD fMRI responses. J Neurosci 35: 5284–5292, 2015. 25834053 10.1523/JNEUROSCI.3787-14.2015 PMC4381001

[B62] Zhang H, Lin SC, Nicolelis MA Spatiotemporal coupling between hippocampal acetylcholine release and theta oscillations in vivo. J Neurosci 30: 13431–13440, 2010. 20926669 10.1523/JNEUROSCI.1144-10.2010 PMC2988451

